# Complicated Crown-Root Fracture Treated Using Reattachment Procedure: A Single Visit Technique

**DOI:** 10.1155/2011/401678

**Published:** 2011-07-18

**Authors:** Akhil Rajput, Sangeeta Talwar, Ida Ataide, Mahesh Verma, Neeraj Wadhawan

**Affiliations:** ^1^Department of Conservative Dentistry and Endodontics, Maulana Azad Institute of Dental Sciences, New Delhi 110002, India; ^2^Department of Conservative Dentistry and Endodontics, Goa Dental College and Hospital, Bambolim, Goa 403202, India; ^3^Maulana Azad Institute of Dental Sciences, New Delhi 110002, India; ^4^Department of Orthodontics, All India Institute of Medical Sciences and Research Centre, New Delhi 110054, India

## Abstract

Complicated crown-root fracture of maxillary central and lateral incisors is common in case of severe trauma or sports-related injury. It happens because of their anterior positioning in oral cavity and protrusive eruptive pattern. On their first dental visit, these patients are in pain and need emergency care. Because of impaired function, esthetics, and phonetics, such patients are quite apprehensive during their emergency visit. Successful pain management with immediate restoration of function, esthetics and phonetics should be the prime objective while handling such cases. This paper describes immediate treatment of oblique crown root fracture of maxillary right lateral incisor with reattachment procedure using light transmitting fiber post. After two and half years, the reattached fragment still has satisfying esthetics and excellent function.

## 1. Introduction

The increased incidence of traumatic injuries to anterior teeth is a consequence of leisure activities, where the most common injuries are crown fractures. These manifestations can vary from simple enamel-dentin fracture to complicated crown-root fracture or root fracture [1]. It has been estimated that about one quarter of the population under the age of 18 sustains traumatic injury in the form of anterior tooth fracture [2]. Out of this, 80% are central incisors and 16% are lateral incisors [3]. This is because of their anterior position and protrusion caused by the eruptive process [4]. Uncomplicated enamel and dentin fracture is most common while those involving crown and root with pulpal exposure constitute only 5–8% of all traumatic injuries [5–8]. A review of published case reports indicate that 85% of traumatized incisors fracture in an oblique fashion from labial to lingual aspect and the fracture line proceeds in apical direction [2]. The type and location of fracture depend upon age of patient, amount of force, and direction of blow [9].

Various methods and techniques were advocated to restore fractured teeth. In the late 60's, temporary and permanent restoration of traumatized teeth in young individuals represented a severe challenge. Methods like resin crowns, stainless steel crowns with or without window preparation, pin-retained inlays, and complex ceramic restorations were in use [10]. All these techniques jeopardized tooth structure and were esthetically unacceptable. Moreover these techniques cannot be used in case of an esthetic emergency [11]. During the 70's, adhesive composite restorations almost became a standard procedure in the treatment of crown fracture in children, adults, and sometimes in elder persons. Other treatment options included porcelain laminate veneers, porcelain fused to metal crowns, and all ceramic crowns.

Acid-etch technique and advances in dentinal adhesives combined to drive a growing minimally invasive treatment philosophy among dentists, as evidenced by a renowned interest in the tooth reattachment procedure [12]. This is possible when tooth fragment is retrieved and preserved after trauma.

The advantages of tooth fragment reattachment over conventional composite restoration are conservatism, favorable wear mechanism, color match of the remaining crown portion, preservation of incisal translucency, maintenance of original tooth contours, preservation of identical occlusal contacts, color stability of the enamel, cost effectiveness, and convenient single visit treatment [13–15]. The real merit of reattachment is the fact that it acts as a transitional restoration for a young patient who may need definitive prosthetic rehabilitations such as direct adhesive veneers or crowns in the event of reattachment failure [16]. 

Nowadays a number of successful reattachment cases and research are reported in literature [12, 16–22]. Still, the prevalence of reattachment procedures is low especially among general dental practitioners. It can be due to either lack of knowledge of such procedures or fear of failure.

## 2. Case Report

**Figure 1:**
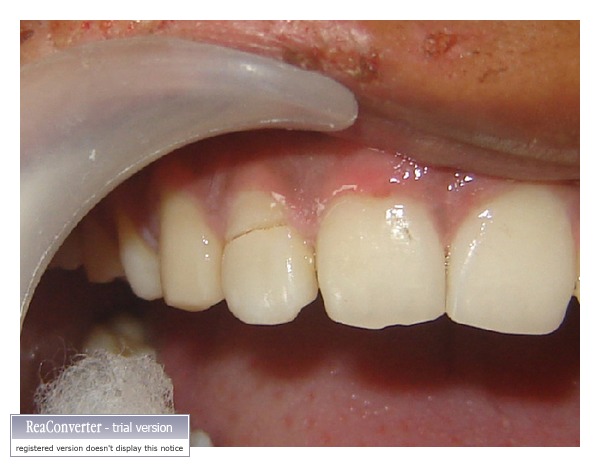
Preoperative view showing complicated crown-root fracture of maxillary right lateral incisor.

A 23-year-old patient reported to the dental hospital after 5 days of trauma and sustained a complicated crown-root fracture to the maxillary right lateral incisor (Figure 1). The fracture line was oblique extending in apical direction from labial to palatal surface. The margin on palatal surface was located about 1.5 mm from the free gingival margin and can be probed easily with a periodontal probe. The fractured fragment was attached to the root but was very mobile. Patient was very apprehensive about his fractured tooth. He was assured, and the condition was explained to the patient. Of the various treatment options explained to the patient, he preferred to retain the fractured fragments. 

**Figure 2:**
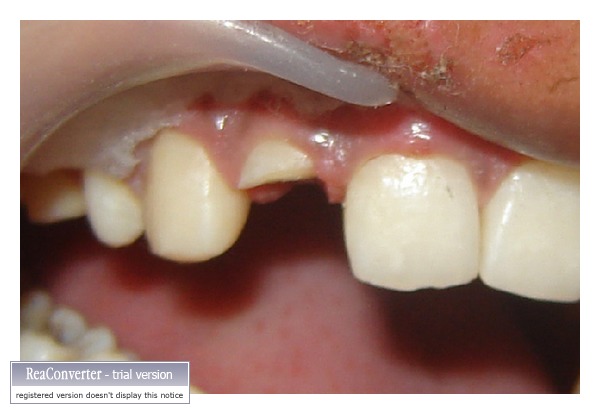
Fracture fragment removed from underlying tooth structure.

**Figure 3:**
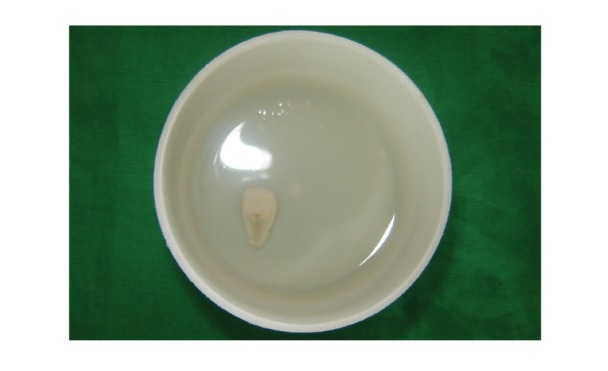
Fractured fragment stored in distilled water till further use.

**Figure 4:**
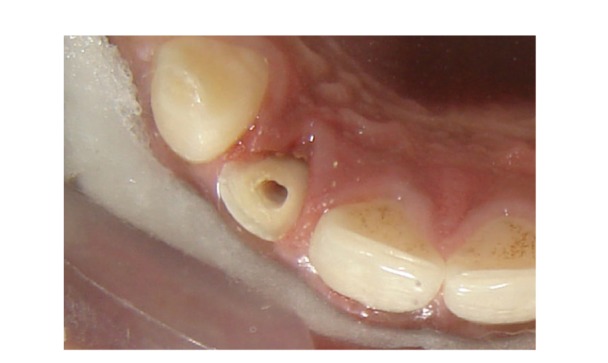
Gelatin sponge was packed on palatal surface in the subgingival area to control any bleeding from that area (mirror image).

**Figure 5:**
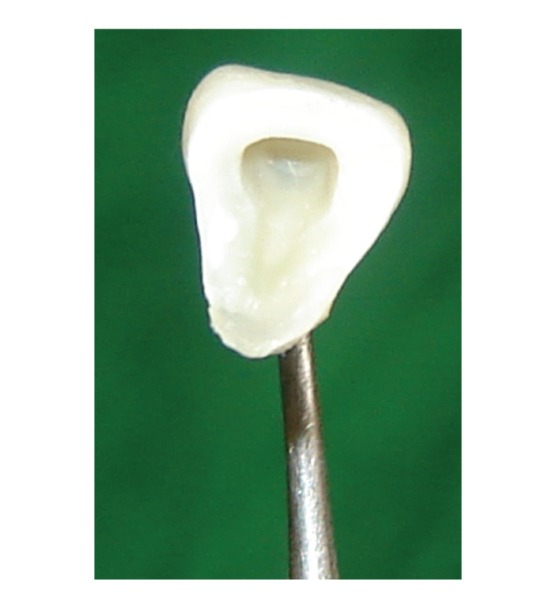
Groove prepared in the fractured fragment.

**Figure 6:**
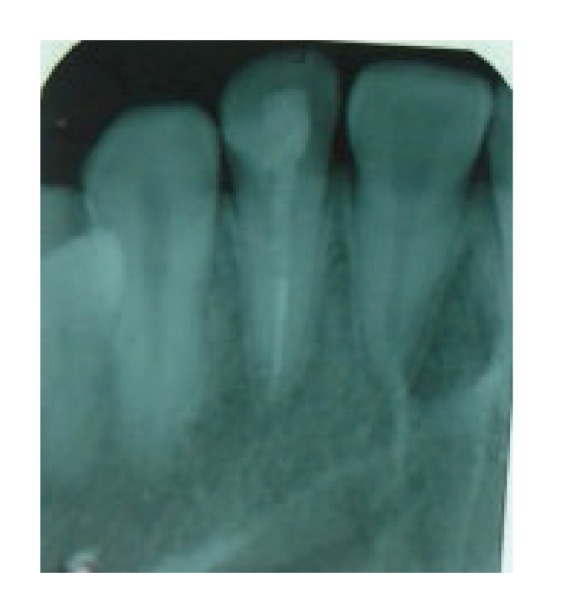
Single visit root canal treatment done and fragment reattached using fiber post and dual cure resin cement.

**Figure 7:**
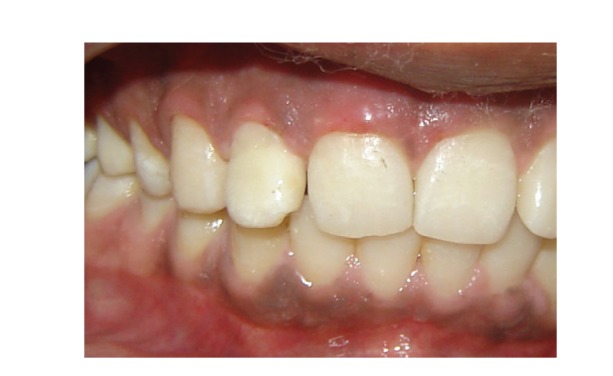
Labial view immediately after restoration of maxillary right lateral incisor.

**Figure 8:**
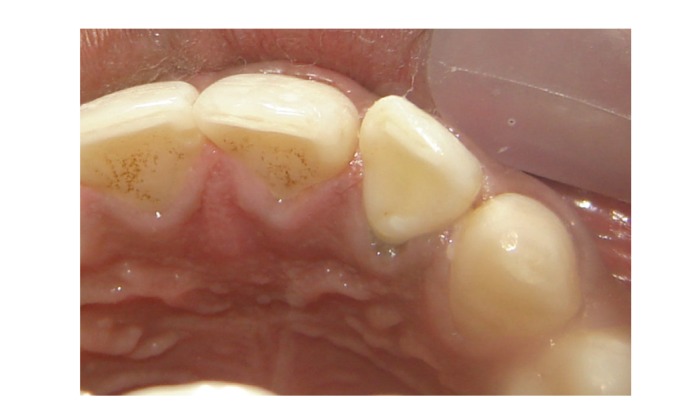
Palatal view immediately after restoration of maxillary right lateral incisor (mirror image).

Since the fractured fragment of the maxillary left central incisor was attached to the underlying root at just one point so it was removed and stored in distilled water to be used at a later stage (Figures 2 and 3). Isolation was achieved using cheek retractor, cotton rolls, and saliva ejector placed in position. Gelatin sponge (Ab Gel, Sri Gopal Krishna Labs, Mumbai, India) was packed on palatal surface in the subgingival area to control any bleeding from that area (Figure 4). Single visit root canal treatment was completed, and the postspace was prepared. A light transmitting fiber post (D.T. Light post, Bisco, Schaumburg, USA) was tried in the canal and cut at the desired length. The fractured fragment was removed from the distilled water and tried on the cut end of fiber post. A groove was made on the fractured fragment so that it fits comfortably on the fractured root without any interference from overlying post (Figure 5). Remnants of pulp tissue from the fractured fragment were removed during this step. Care was taken not to remove excess dentin as it can alter the final esthetic appearance of the tooth. Once the desired fit was confirmed, it was again stored in distilled water. After acid etching with 37% phosphoric acid (Total etch, Ivoclar Vivadent AG, Bendererstrasse, Liechtenstein) of root canal, dual cure bonding agent (Prime and Bond NT dual cure, DENTSPLY Caulk, Milford DE) was applied as per the manufacturer's instructions, and the post was cemented with the help of dual cure resin cement (Calibra esthetic resin cement, DENTSPLY Caulk, Milford DE). Any excess cement oozing out of canal was removed with cotton applicator tips as it can alter the fit of the fragment. It was then light cured (QHL75 Curing Light, DENTSPLY, Addlestone, Surrey) for 40 sec. Gelatin sponge was then removed, and exposed root surface and fractured fragment were acid etched simultaneously. Groove in the fractured fragment was filled with dual cure resin cement, and the exposed fiber post was also luted with the same. The fragment was repositioned and cured for 40 sec. from palatal, labial, and incisal surfaces (Figure 6). Since the fracture line was visible on the labial surface, a groove was made app. 0.3 mm deep, extending app. 1.5 mm incisally and gingivally from the fracture line. It was then restored with Nanocomposite (Filtek Z350 Universal Restorative, 3M ESPE, St. Paul, Minn, USA) (Figures 7 and 8). Finishing and polishing was done using Sof-Lex polishing system (Sof-Lex Extra Thin Contouring and Polishing Discs, 3M ESPE, St. Paul, Minn, USA). Orthodontic extrusion of same tooth was planned after two week so as to bring the palatal fracture line supragingival and seal any defect, if present. But, the patient was so satisfied with the results after two weeks that he refused any further treatment. 

**Figure 9:**
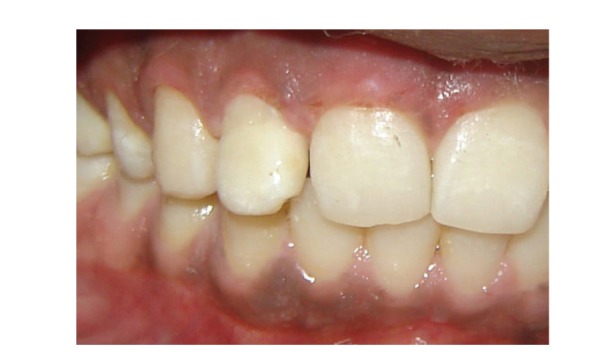
Labial view after two and half years.

**Figure 10:**
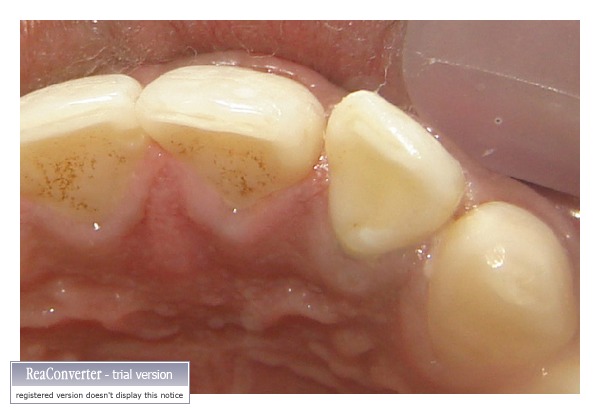
Palatal view after two and half years (mirror image).

**Figure 11:**
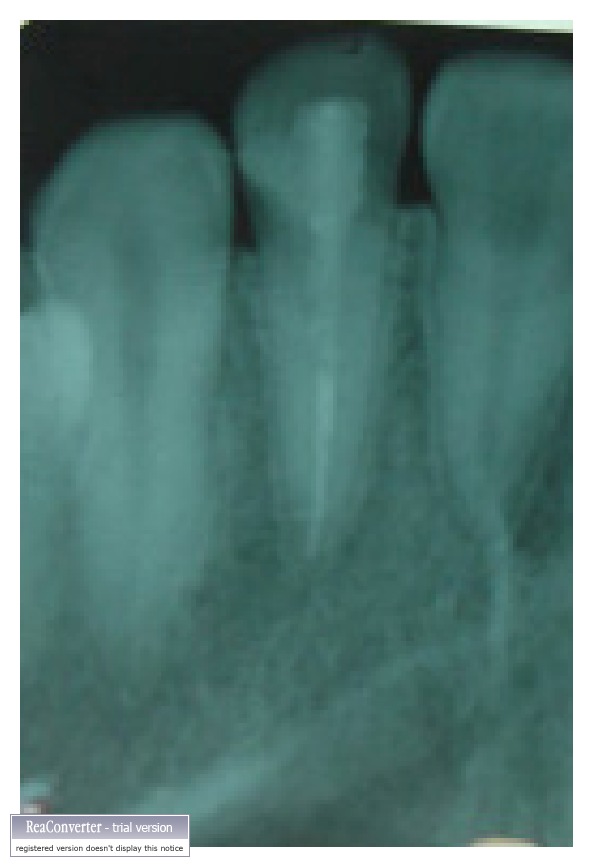
Radiographic appearance after two and half years.

After two and half years, the treated tooth has satisfying excellent esthetics and function (Figures 9 and 10). There was no mobility of any of the fragments, and the periodontal status in relation to maxillary right lateral incisor was satisfactory (no periodontal pockets with normally contoured palatal gingiva). Radiographic examination reveals satisfactory healing, and no discoloration was evident on clinical examination (Figure 11).

## 3. Discussion

A patient with fractured anterior teeth usually reports with pain and is emotionally upset about his or her appearance [23]. Quick restoration of the esthetic appearance and relief of discomfort for these patients within a single appointment by preserving the natural tooth structure may lead to a positive emotional and social response from the patient. The technique described in this paper is simple, quick and economic as compared to other more invasive procedures. A number of case reports explain the successful reattachment of an uncomplicated tooth fracture cases. This paper signifies that the fragment can be used even if the fracture is complicated but if the margins are accessible. Isolation is the key to success in such cases. Rubber dam was not possible in this case, but adequate isolation was achieved using cotton rolls, cheek retractor, and gelfoam. Orthodontic extrusion, if required, can be planned once the other injuries of face have healed. It is to expose the fracture line to oral cavity and seal any visible defect at the margin. As with all traumatic injuries, followup is of critical importance. The patient should be followed for 3, 6, 12 months and yearly for 5 years [24]. Esthetics, tooth mobility, and periodontal status should be confirmed both clinically and radiographically at these follow-up visits.

## References

[1] Holan G, Shmueli Y (2003). Knowledge of physicians in hospital emergency rooms in Israel on their role in cases of avulsion of permanent incisors. *International Journal of Paediatric Dentistry*.

[2] Murchison DF, Burke FJT, Worthington RB (1999). Incisal edge reattachment: indications for use and clinical technique. *British Dental Journal*.

[3] Andreasen JO, Ravn JJ (1972). Epidemiology of traumatic dental injuries to primary and permanent teeth in a Danish population sample. *International Journal of Oral Surgery*.

[4] Andreasen JO, Andreasen FM (1994). *Textbook and Color Atlas of Traumatic Injuries to the Teeth*.

[5] Wood EB, Freer TJ (2002). A survey of dental and oral trauma in south-east Queensland during 1998. *Australian Dental Journal*.

[6] Liew VP, Daly CG (1986). Anterior dental trauma treated after-hours in Newcastle, Australia. *Community dentistry and oral epidemiology*.

[7] Martin IG, Daly CG, Liew VP (1990). After-hours treatment of anterior dental trauma in Newcastle and western Sydney: a four-year study. *Australian dental journal*.

[8] Andreasen JO (1981). *Traumatic Injures of the Teeth*.

[9] Andreasen JO (1970). Etiology and pathogenesis of traumatic dental injuries. A clinical study of 1,298 cases. *Scandinavian Journal of Dental Research*.

[10] Buonocore MG, Davila J (1973). Restoration of fractured anterior teeth with ultraviolet-light-polymerized bonding materials: a new technique. *The Journal of the American Dental Association*.

[11] Andreasen JO (2001). Buonocore memorial lecture. Adhesive dentistry applied to the treatment of traumatic dental injuries. *Operative Dentistry*.

[12] Arhun N, Ungor M (2007). Re-attachment of a fractured tooth: a case report. *Dental Traumatology*.

[13] Reis A, Loguercio AD, Kraul A, Matson E (2004). Reattachment of fractured teeth: a review of literature regarding techniques and materials. *Operative Dentistry*.

[14] Baratieri LN, Ritter AV, Monteiro Júnior S, de Mello Filho JC (1998). Tooth fragment reattachment: an alternative for restoration of fractured anterior teeth. *Practical Periodontics and Aesthetic Dentistry*.

[15] Reis A, Loguercio AD (2004). Tooth fragment reattachment: current treatment concepts. *Practical Procedures &amp; Aesthetic Dentistry*.

[16] Maia EAV, Baratieri LN, de Andrada MAC, Monteiro S, de Araujo EM (2003). Tooth fragment reattachment: fundamentals of the technique and two case reports. *Quintessence International*.

[17] Turgut MD, Gönül N, Altay N (2004). Multiple complicated crown-root fracture of a permanent incisor. *Dental Traumatology*.

[18] Nogueira Filho Gda R, Machion L, Teixeira FB, Pimenta LA, Sallum EA (2002). Reattachment of an autogenous tooth fragment in a fracture with biologic width violation: a case report. *Quintessence International*.

[19] Koparal E, Ilgenli T (1999). Reattachment of a subgingivally fractured central incisor tooth fragment: report of a case. *Journal of Clinical Pediatric Dentistry*.

[20] Baratieri LN, Monteiro Júnior S, Cardoso AC, de Melo Filho JC (1993). Coronal fracture with invasion of the biologic width: a case report. *Quintessence International*.

[21] Ludlow JB, LaTurno SA (1985). Traumatic fracture–one-visit endodontic treatment and dentinal bonding reattachment of coronal fragment: report of case. *The Journal of the American Dental Association*.

[22] Öz IA, Haytaç MC, Toroğlu MS (2006). Multidisciplinary approach to the rehabilitation of a crown-root fracture with original fragment for immediate esthetics: a case report with 4-year follow-up. *Dental Traumatology*.

[23] Mader C (1978). Restoration of a fractured anterior tooth. *The Journal of the American Dental Association*.

[24] Rajput A, Ataide I, Fernandes M (2009). Uncomplicated crown fracture, complicated crown-root fracture, and horizontal root fracture simultaneously treated in a patient during emergency visit: a case report. *Oral Surgery, Oral Medicine, Oral Pathology, Oral Radiology and Endodontology*.

